# Structure-Based Discovery of ABCG2 Inhibitors: A Homology Protein-Based Pharmacophore Modeling and Molecular Docking Approach

**DOI:** 10.3390/molecules26113115

**Published:** 2021-05-23

**Authors:** Minh-Tri Le, Viet-Nham Hoang, Dac-Nhan Nguyen, Thi-Hoang-Linh Bui, Thien-Vy Phan, Phuong Nguyen-Hoai Huynh, Thanh-Dao Tran, Khac-Minh Thai

**Affiliations:** 1 Faculty of Pharmacy, University of Medicine and Pharmacy at Ho Chi Minh City, Ho Chi Minh City 100000, Vietnam; leminhtri@ump.edu.vn (M.-T.L.); hoangvietnham@gmail.com (V.-N.H.); michael.dacnhan@gmail.com (D.-N.N.); bthlinh.duoc16@ump.edu.vn (T.-H.-L.B.); ptvy@ntt.edu.vn (T.-V.P.); huynhnguyenhoaiphuong@gmail.com (P.N.-H.H.); daott@ump.edu.vn (T.-D.T.); 2 School of Medicine, Vietnam National University Ho Chi Minh City, Ho Chi Minh City 100000, Vietnam; 3 Department of Pharmacy, Lac Hong University, Bien Hoa 810000, Vietnam; 4 Department of Pharmacy, Nguyen Tat Thanh University, Ho Chi Minh City 100000, Vietnam

**Keywords:** ABCG2 inhibitors, protein reverse transport pump, homology protein, pharmacophore models, molecular docking

## Abstract

ABCG2 is an ABC membrane protein reverse transport pump, which removes toxic substances such as medicines out of cells. As a result, drug bioavailability is an unexpected change and negatively influences the ADMET (absorption, distribution, metabolism, excretion, and toxicity), leading to multi-drug resistance (MDR). Currently, in spite of promising studies, screening for ABCG2 inhibitors showed modest results. The aim of this study was to search for small molecules that could inhibit the ABCG2 pump. We first used the WISS MODEL automatic server to build up ABCG2 homology protein from 655 amino acids. Pharmacophore models, which were con-structed based on strong ABCG2 inhibitors (IC_50_ < 1 μM), consist of two hydrophobic (Hyd) groups, two hydrogen bonding acceptors (Acc2), and an aromatic or conjugated ring (Aro|PiR). Using molecular docking method, 714 substances from the DrugBank and 837 substances from the TCM with potential to inhibit the ABCG2 were obtained. These chemicals maybe favor synthesized or extracted and bioactivity testing.

## 1. Introduction

During the last decades, multi-drug resistance (MDR) is one of the main problems that challenge clinical treatment, especially chemotherapy for various cancers. One of the most crucial agents of drug resistance is related to the ABC membrane protein reverse transport pumps. ABCG2 was described by Dean M. et al. as the second member of the G subfamily, a subfamily in the ABC family of proteins [1]. The protein, also known as BCRP, plays a role as a toxic removal channel, in which stranger substances, including medicines, are metabolized and excreted out of cells. It is presented throughout the body, in different tissues such as intestines, liver, kidneys, and cancerous tissue. Consequently, drug bioavailability is an unexpected change and negatively influences the ADMET (absorption, distribution, metabolism, excretion, and toxicity) properties of many medications as well as drug-like molecules. In cancer cells, an increase in ABCG2 expression was associated with therapy failure. Therefore, the discovery of ABCG2 inhibitors could improve the bioavailability of the desired drug, minimize multidrug resistance, and result in more effective therapy.

There are many studies regarding the structure and effects of ABCG2 inhibitors. According to some studies, the hydrophobic properties in flavonoids and the derivatives of fumitremorgin C are supposed to be important agents for ABCG2 inhibitory activity [2,3,4]. In addition, a study on tariquidar and propafenon derivatives showed that a flat ring structure enhances ABCG2’s inhibitory activity, which can be seen in two strong ABCG2 inhibitors namely purvalanol A and WHI-P180 (3-[(6,7-dimethoxyquinazolin-4-yl) amino] phenol) [5]. Despite of these promising results, screening for ABCG2 inhibitors is still limited because of the low resolution 3D-structure of this protein. Regarding some studies on ABCG2 inhibitors, the potential candidates were Sitravatinib, BMS-599626 and PZ-39 with various mechanism [6,7,8]. While virtual screening from DrugBank databases revealed cisapride and roflumilas could well inhibit BCRP on PLB985 expressed cell [9], SAR and QSAR studies showed dissimilar outcomes [2,10,11].

In the study, in silico models was built for screening ABCG2 pump inhibitors. The pharmacophore model was created based on ligands which are substances with a strong inhibitory activity (IC_50_ < 1 μM), while molecular docking model was contributed on ABCG2 homology protein model, which was built up from 655 amino acids using WISS MODEL automatic server. We use two large structure libraries for in silico screening, which are 15,464 substances from the DrugBank database [12] and 57,424 substances from the Traditional Chinese Medicine Collection (TCM) [13]. A pharmacophore was firstly used to find out the lead compounds, and then these ligands were docked in an ABCG2 homology protein model to examine protein–ligand interaction. Substances with good results after screening throughout this process may favor synthesis or extraction and bioactivity testing.

## 2. Results

### 2.1. Pharmacophore Model

**Figure 1:**
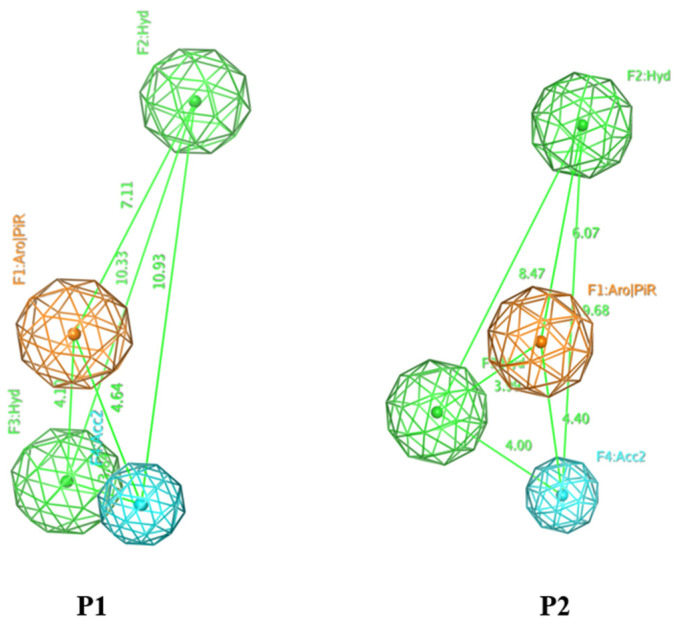
The spatial position and the distance of two models (**P1**) and (**P2**).

From 15 strong inhibitors (Supplementary Table S1), the study has built two four-point pharmacophore models, P1 and P2, with accuracies of 15/15 and 14/15, respectively, including 2 hydrophobic centers, 1 aromatic ring, and 1 hydrogen bonding acceptor (Table 1). The spatial position and the distance of the models P1 and P2 are described in Figure 1, and these two models were preliminarily estimated with a decoy set (Table 2).

The results showed that both models were not selective even though their sensitivities were over 90 percent, but their low specificity and index E together with GH score <0.5 indicated that the screening models were not good. This issue was solved by the Pharmacophore Query Editor tool in MOE 2015.10. Before overcoming this limitation, the importance is seeking the relatively selective query. The activity value of overlap parameter is 0.9 while building automatic pharmacophore, all queries are generated with the accuracy of nearly 10,000. With this accuracy, the queries meet 100% both weak-medium binding set and non-binding set. Implementing to overlap 118 substances with the lowest IC_50_ value from the strong inhibitory set. As the number of substances in the training set is large, with the diversity of structures (118 substances), finding a query that satisfies over 90 percent of the constructive set, but does not satisfy the non-bonding set is very complicated. Therefore, building the activity of the overlap parameter decreasing to 0.8 for the purpose of generating the model that was more selective for weak-binding and non-binding set in spite of not achieving the high accuracy. The two obtained models aligning with 118 strong inhibitors ABCG2 were called P3 and P4 (Table 3). 

**Figure 2:**
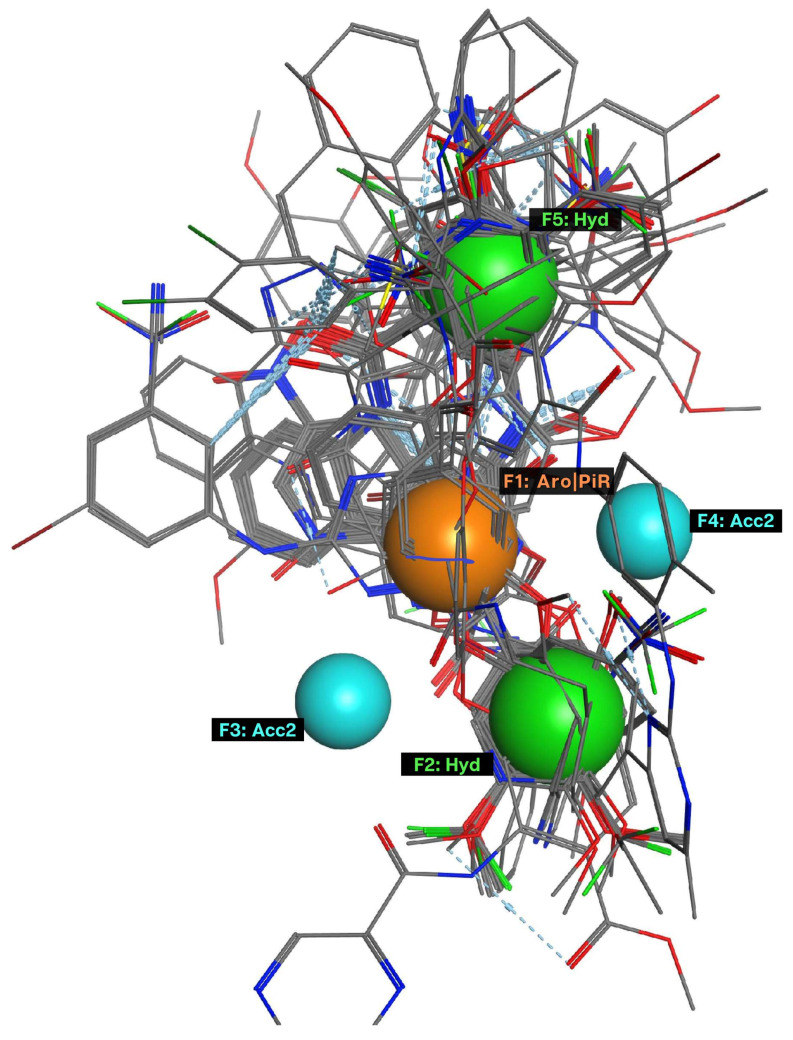
A hydrophobic factor was added to four-point pharmacophore model.

The obtained model P3 had the high repeatability, 107 over 118 molecules constituting 90.68%. In addition, after alignment, it remained one more position for adding the fifth pharmacophore factor as described in Figure 2. This model became a five-point pharmacophore model (P5), including 2 hydrophobic groups (Hyd, H), an aromatic ring or π conjugated ring (Aro/PiR), and 2 hydrogen bonding acceptor (Acc2). Thus, the models would comprise of 3 hydro-phobic features, 2H and 1 Aro/PiR. Evaluation results of 5-point pharmacophore model P5 are shown in Table 4. The results show that this model is relatively selective on strong inhibitors. 

**Figure 3:**
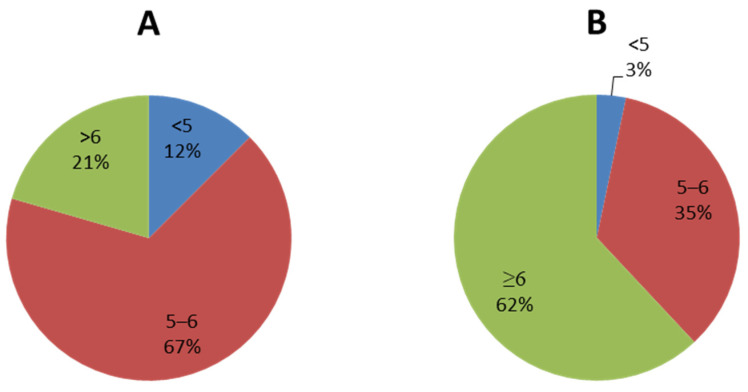
Binding sites determined by Site Finder Distribution of substances depended on biological activity in the satisfied (**B**) or unsatisfied pharmacophore model set (**A**).

**Figure 4:**
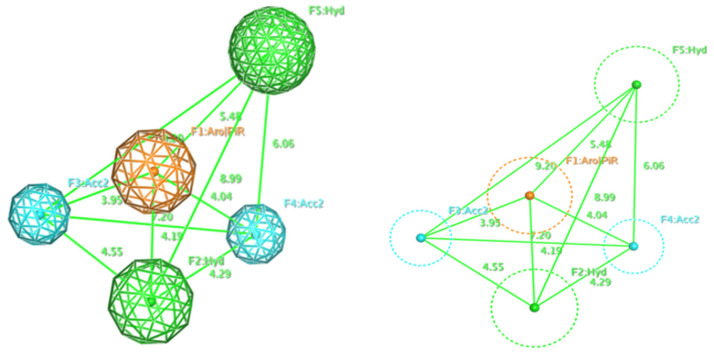
Factors of five-point pharmacophore model. F3, F4 (**the blue**): hydrogen bonding acceptors (Acc2); F2, F5 (**the green**): hydrophobic groups (Hyd); F1 (**the orange**): an aromatic ring or π conjugated ring (Aro|PiR).

Conducting a test of pharmacophore model RHHaa_1 on the known biological activity set. The trial set had 375 substances, including 50 inactive substances and 325 active substances. There were 213 over 325 substances satisfying the pharmacophore model (Supplementary Table S2), occupying 65.54%. Distribution of substances depended on biological activity (strong, weak, inactive) in the satisfied or unsatisfied pharmacophore model set (Figure 3). Substances with strong biological activity (pIC_50_ ≥ 6) satisfying the pharmacophore model accounted for 62%, which is almost 3 times higher than in the set of unsatisfied pharmacophore model substances (62/21). Meanwhile, the ratio of substances with biological activity below 6 was 2 times lower (38/79), proving that the pharmacophore model was selective on strong inhibitors of ABCG2. The factors of the pharmacophore model were described in Figure 4. Therefore, the P5 model will be used to screen for strong ABCG2 inhibitors.

### 2.2. Molecular Docking Model

#### 2.2.1. Homology Model

**Figure 5:**
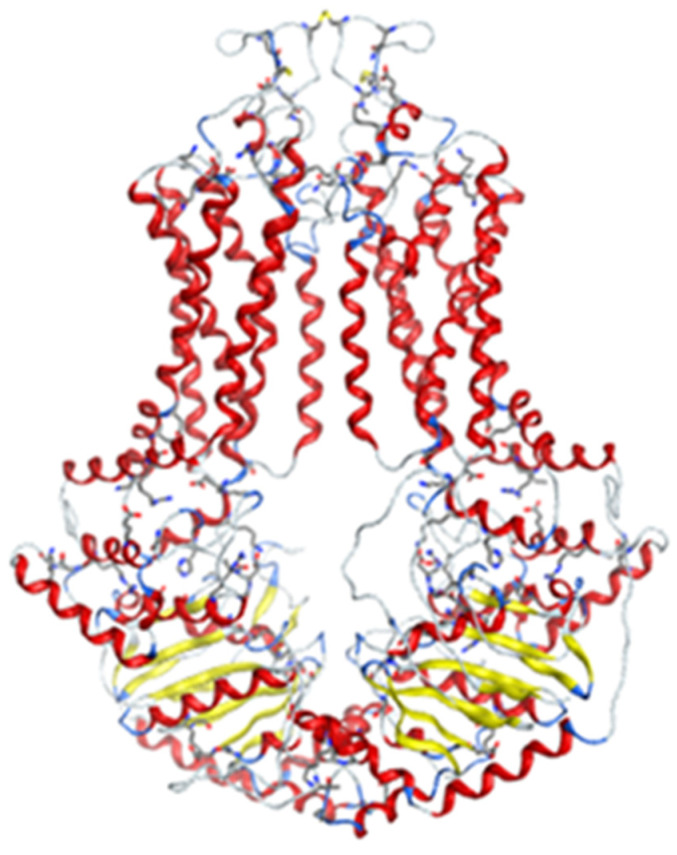
Homology model of ABCG2.

**Figure 6:**
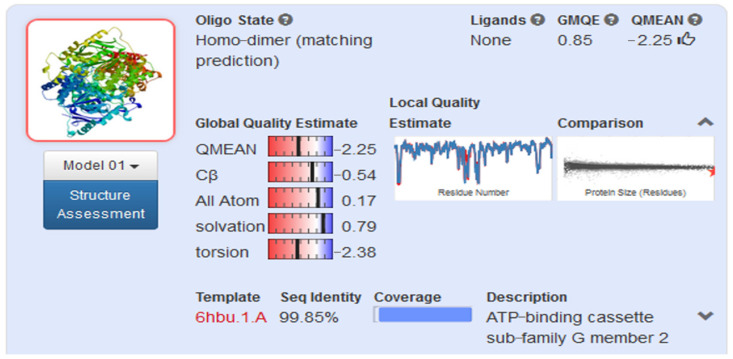
Parameters for evaluating homology model from SWISS-MODEL site.

Homology model was created from 655 amino acids of ABCG2 in www.uniprot.org (accessed on 17 September 2019) by SWISS-MODEL server [14] (Figure 5). This model was built from the model 6hbu.1.A, which was an electron microscopy model [15]. The model structure consisted of two protein chains, each of which forms half of the ABCG2 transport pump. Each half-channel included 6 integral membrane fragment. A short fragment of ATP-binding was located inside the cell, while the area outside the cell obtained disulfide bonds. In general, this structure was similar with others that had been built by electron microscope photography [16]. The model’s evaluation is shown in Figure 6. The GMQE (Global Model Quality Estimation) value of the homology model was 0.85, while the QMEAN (Qualitative Model Energy Analysis) value was −2.25 (above −4). Therefore, this model could be used to construct molecular docking.

**Figure 7:**
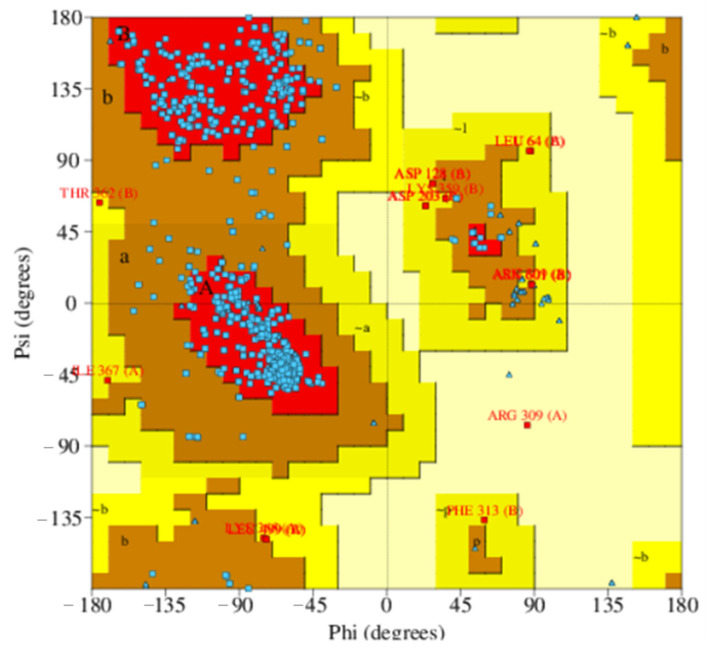
Ramachandran plot of the best ABCG2 homology model, in which the most favored regions, the additional allowed regions, the generously allowed regions and the disallowed regions were labeled [A, B, L]; [a, b, l, p]; [~a, ~b, ~l, ~p] and [XX], respectively.

In a Ramachandran map, the most favorable region was illustrated in the red area, whilst the less favorable regions were showed in progressively lighter tones (Figure 7). Among 1104 non-glycine and non-proline residues of the homology protein, 91.1% (1006 residues), 7.3% (81 residues), 1.4% (16 residues), and 0.1% (1 residue) were, respectively, found in the most favored regions [A,B,L]; the additional allowed regions [a, b, l, p]; the generously allowed regions [~a, ~b, ~l, ~p]; and the disallowed regions [XX]. These figures were also similar in the Ramachandran plot of the ATP-binding cassette sub-family G member-2 sample model (6hbu.1.A). Moreover, the statistics indicated that the selected homology model had more than 90% of residues in the core regions [A, B, L], meaning this high quality model was appropriate for further in silico screening research.

**Figure 8:**
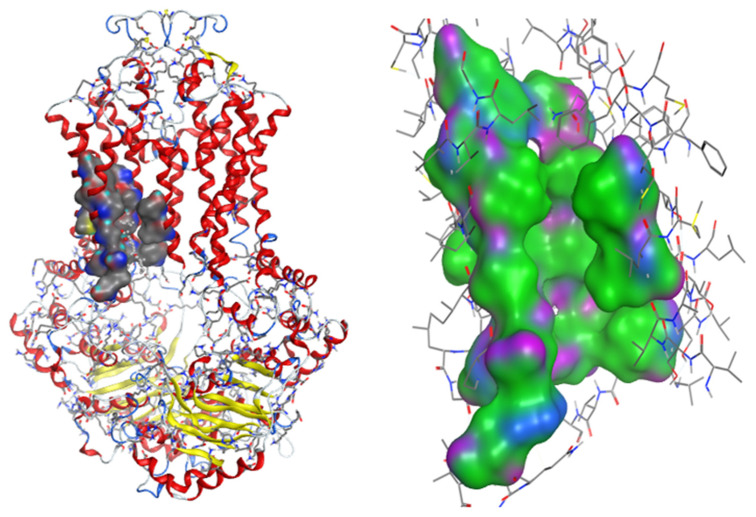
The binding site of the inhibitor on ABCG2. The green area represents the hydrophobic zone, the blue area represents the slightly polarized zone, the pink area represents the hydrophilic zone.

Using the Site Finder search tool and docking with Lead IT, the binding site of ABCG2 inhibitors was determined in Figure 8, which consisted of 20 amino acids belonging to two chains of ABCG2 pump: Chain A included Phe 182, Asn 391, Gln 393, Ala 394, Ala 397, Gln 398, Val 401, Thr 402, Gln 437, Ser 440, Ser 441, Ser 443, Glu 444, Glu 446, Met 481, Arg 482 and Pro 485; Chain B included Val 534, Leu 539 and Ile 543. In which the amino acids Agn391, Gln 393, Gln 398, Thr 402, Gln 437, Ser 440, Ser 441, Ser 443, Glu 444, Glu 446, and Arg 482 were po-lar amino acids form the hydrophilic region in the binding site. Meanwhile, amino acids such as Phe182, Ala 394, Ala 397, Val 401, Met 481, Prolin 485, Val 534, Leu 539, and Ile 543 made up the hydrophobic region in the binding site. This binding site also matched the one identified in previous research by Laura et al. [17].

#### 2.2.2. Docking Models

**Figure 9:**
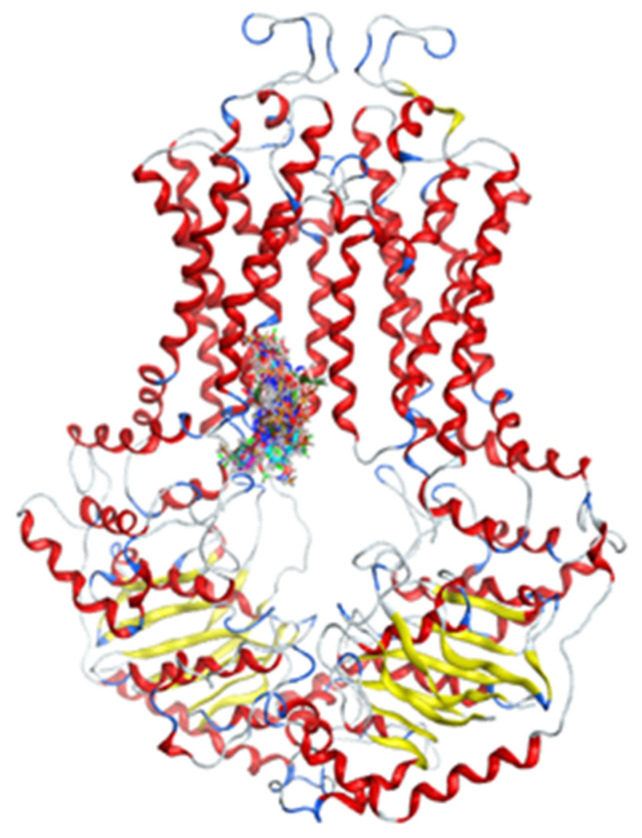
Binding sites of 325 substances on the ABCG2 protein.

The docking scores of 15 strong inhibitors ranged from −5.15 KJ·mol^−1^ to −22.74 KJ·mol^−1^. In the binding pocket, these substances could bind well and interact with important amino acids (Table 5). Besides, other 325 substances, including 155 strong inhibitors and 170 weak ones, were also docked into the binding site. Figure 9 shows that all 325 substances were successfully docked into ABCG2 protein with docking score varied from −4.1 to −28.2 KJ·mol^−1^ (Supplementary Table S2). 

**Figure 10:**
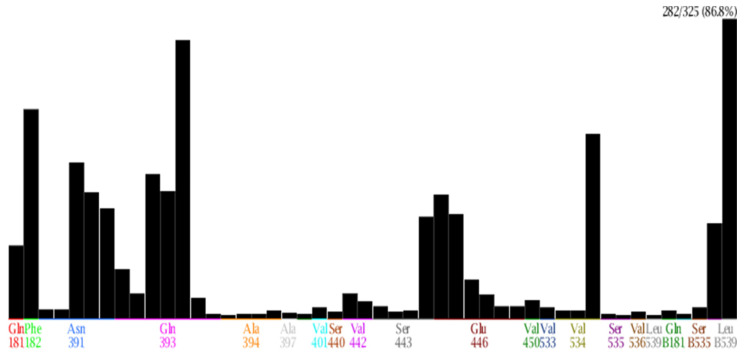
The frequency of amino acids interacting in the binding site.

By using PLIF tool in MOE, the frequency of amino acids interacting with amino acids results could be seen in Table 6 and Figure 10, in which Phe 182, Asn 391, Gln 393, Glu 446, Ser 443, Val 533, Val 534, Val536, and Leu 539 were determined as important amino acids with a high frequency of interaction. As can been seen from docking models, a good bonding ligand requires the following: The hydrogen-bond donors and acceptors that can combine with the polar amino acids (Asn 391, Gln 393, Glu 446); the hydrophobic group that can go deep into the binding site to interact with hydrophobic amino acids (Ser 443, Val 533, Val 534, Val536, Leu 539) and the interaction between aromatic ring and Phe182 in the binding site. 

**Figure 11:**
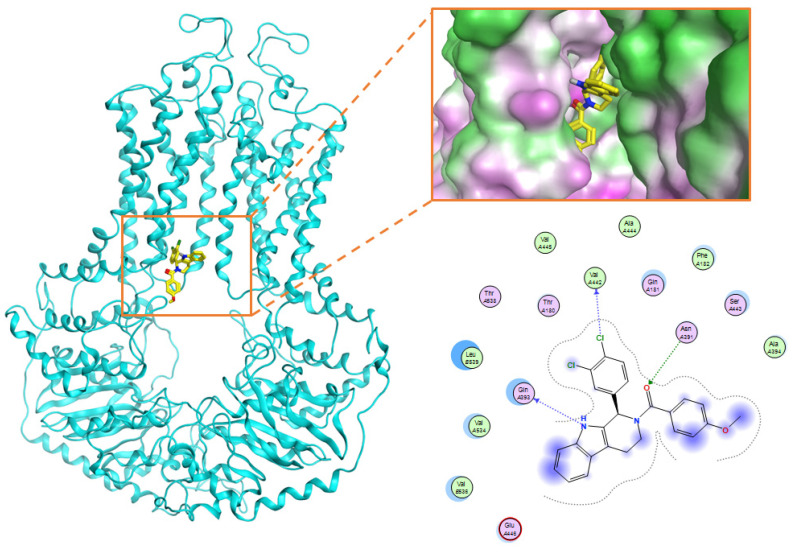
Binding site and interactions of JMC_2016_59_6121_51; DS = −16.14 KJ·mol^−1^.

**Figure 12:**
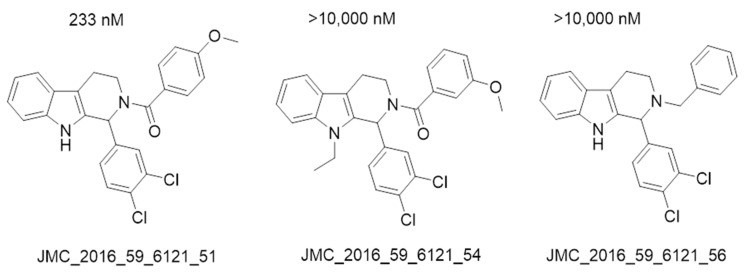
Structure and biological activity of tetrahydroxy-β-carbolin derivatives.

The docking result of a derivative of Fumitremorgin C (Ko143) in the binding site shows that this structure does not go deep into the binding site. The tertbutyl group was a donor in a hydrogen bond with Gln 393 and hydrophobic interacted with Ala 397, Val 536, Leu 539 in 2 regions. Isopropyl group that interacted with Phe 182 played an important role in this group of compounds. As the case of tetrahydroxy-β-carbolin derivative, JMC_2016_59_6121_51 (Figure 11), the -NH- group on the carbolin skeleton interacted with Gln 393, and the C=O group interacted with Asn 391, which were two important functional groups in this group of compounds. This result was consistent with the biological activity test of tetrahydro-β-carbolin group when ethylated -NH- group on this molecule, the biological activity decreased significantly as shown in Figure 12. 

**Figure 13:**
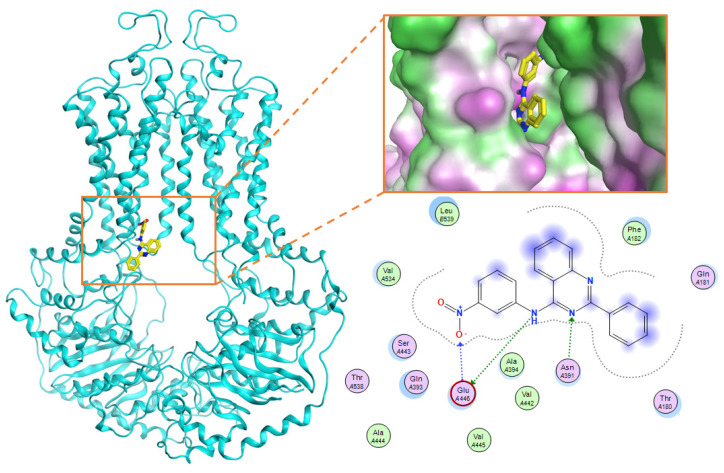
Binding site and interactions of BMC_2013_21_7858_20; DS = −23.51 KJ·mol^−1^.

In the Chalcon derivatives, the hydrogen bonds (donor or acceptor) of Asn 391 and Gln 393 with 2 methoxy groups on ring A played a vital role. The hydrophobic groups of ring B interacted with the hydrophobic amino acids (Leu 539, Ala 397, Val 536). This was consistent with the bioactive test. The molecule JMC_2013_67_115_17 belongs to the group of Flavone and benzoflavone derivatives, the oxygen atoms of the C ring acted as a hydrogen bond acceptor with Glu 393. The hydrophobic groups such as prenyl, methoxyl on the A ring of the chromone could have good activity by interacting with the hydrophobic amino acids of the binding site such as Leu539, Ala397, and Val536. In addition, the hydrogen bond acceptor of the ring B (such as the methoxyl group) could be a way to increase the interaction. The docking score of Quinazoline derivatives group was quite low, showing good interaction with the binding site. In the structure BMC_2013_21_7858_20 (Figure 13), quinazoline had 2 nitrogen atoms as hydrogen bond acceptors with Gln181, Asn391, and Gln 393; the secondary amine of quinazoline with phenyl group may donate hydrogen to have hydrogen bond with Glu 446 and Gln 393. The nitrite group, which was involved in the hydrogen bond acceptor with Glu446, can be replaced by the hydrogen bond donor or acceptor. The phenyl group was required to create hydrophobic interactions with amino acids such as Phe181. The result was better when added to the methoxy group. 

In the Tariquidar derivatives, the smallest docking score substances in the groups, the inhibitor molecule went deep into the binding site. CMC_2012_7_650_nilotinib had a docking score of −26.408 KJ·mol^−1^ and hydrogen-acceptor interactions of the amino acids Asn 391 and Gln 393 still played an important role.

**Figure 14:**
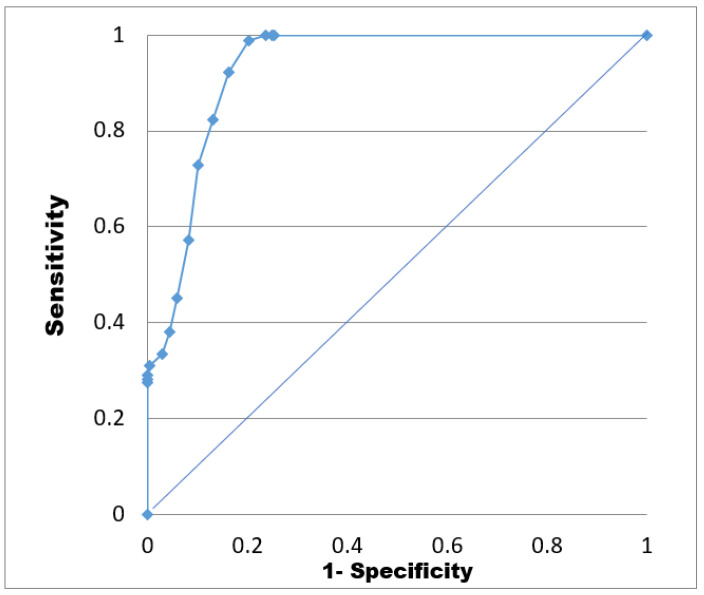
Receiver operating characteristic (ROC) curves of homology models.

There were 927/950 substances of the decoy set that were successfully docked into the protein, with the docking scores ranging from −23.37 KJ·mol^−1^ to +15.6 KJ·mol^−1^. Meanwhile, all 325 active substances were docked to the binding site with the docking scores ranged between −28.34 KJ·mol^−1^ and −4.12 KJ·mol^−1^. As a potential inhibitor should have docking score lower than −20 KJ·mol^−1^, the AUC were 0.92 and the EF value were 3.62 (Table 7) (Figure 14). 

### 2.3. In Silico Screening

The docking and pharmacophore models were correlated. Hydrogen-bond acceptor factors F2: Acc2 and F3: Acc2 on the pharmacophore model correspond to the amino acid regions Asn 391, Gln 393, and Glu 446, which were polar amino acids with the ability to form hydrogen bonds. The 2 hydrophobic elements F5: Hyd and F1: Aro|PiR correspond to the hydrophobic region created by 3 amino acids: Ala 397, Val 536, and Leu 539. The hydrophobic region F2: Hyd corresponds to the interaction area of the Phe 181.

**Figure 15:**
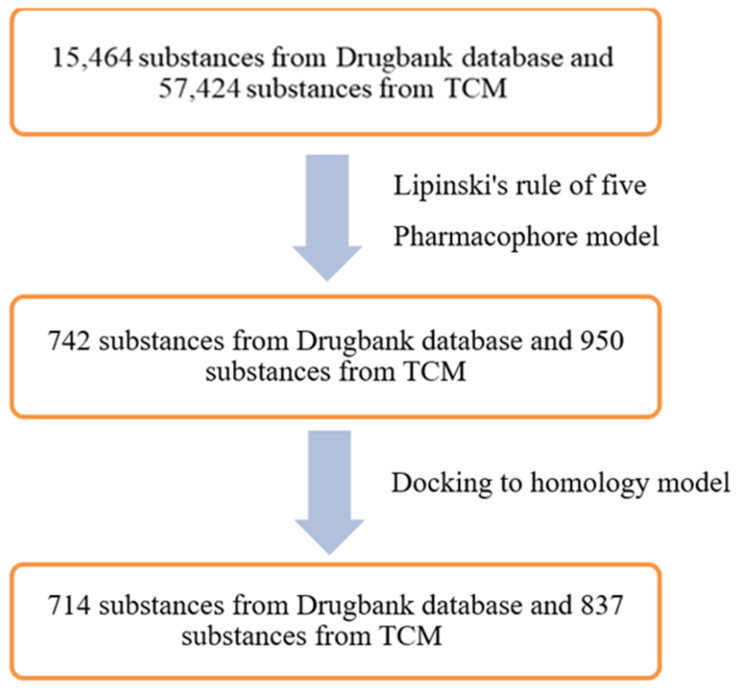
Screening scheme of ABCG2 inhibitors.

The databases used for screening included 57,724 compounds from the TCM (Traditional Chinese Medicine) [13] and 15,464 compounds from the DrugBank database [12]. These compounds were formulated by MOE 2015.10 software, then screened with pharmacophore model RHHaa_1 with a “druglike” filter to eliminate substances not meeting the Lipinski’s “Rule of five”. The results obtained 742 compounds belonging to the DrugBank database and 2743 com-pounds belonging to the TCM database. As the number of the TCM database’s compounds was very large, the filtration of compounds with an 0.7 of RMSD compared to the pharmacophore model was conducted to yield 950 compounds. These compounds were docked into the binding site as described in the research method to evaluate the results. The number of compounds from the DrugBank and TCM database successfully docked into the binding pocket were 714 and 837, respectively. The screening steps are described in Figure 15.

Based on docking score values, selected compounds with inhibitory potential for ABCG2 are listed in Table 8. Among the compounds selected from DrugBank, there were chemicals containing pyrimidine derivative such as 1, 3, 5, tetrahydro-β-carbolin frame derivative as number 10 or bisphenyl frame as substance 9. Among the compounds screened from the TCM set, most of the chemicals belonged to the flavonoid structure or the purine backbones. These compounds were presented in many plants such as: compound 11 is Rosmarinic acid, which is abundant in the Lamiaceae family, while compound 19 is 6-Prenyleriodictyol, which is abundant in the *Glycyrrhiza uralensis.*

## 3. Discussion

In this research, pharmacophore models were constructed based on substances with strong ABCG2 inhibitory activity (IC_50_ ≤ 1 μM) with a sensitivity of 73.33% and a specificity of 92.95%. From these models, it is possible to indirectly infer molecular properties of ABCG2 inhibitors and the interaction characteristics of compounds with the target.

The potent pharmacophore models consist of 5 features including 2 hydrophobic groups (Hyd), 1 aromatic ring (Aro|PiR) and 2 hydrogen bonding acceptors (Acc2). The proportion of hydrophobic groups is 3/5, consistent with studies on hydrophobicity of the binding site. The hydrophobic feature (including aromatic ring and hydrophobic groups), the hydrogen bonding acceptors and the distances between features have similarities with many previous studies.

The protein homology model of ABCG2 was built from 655 amino acids by server SWISS MODEL with GMQE value = 0.85 (the closer to 1, the more similar to the original model), the QMEAN value is −2.25 > −4. The model is eligible to conduct molecular docking model. 

The research has also identified the binding regions of ABCG2 inhibitors with important amino acids such as Gln 181, Phe 182, Asn 391, Gln 393, Glu 446, Ser 443, Val 533, Val 534, Val 536, and Leu 539. Asn 391, Gln 393, and Glu 446 form hydrogen bonds with inhibitors, and these amino acids correspond to hydrogen bonding acceptors in the pharmacophore model. The remaining amino acids form a binding site with hydrophobic regions. 

The binding site consists of chain A (Phe 182, Asn 391, Gln 393, Ala 394, Ala 397, Gln 398, Val 401, Thr 402, Gln 437, Ser 440, Ser 441, Ser 443, Glu 444, Glu 446, Met 481, Arg 482, and Pro 485) and chain B (Val 534, Leu 539, and Ile 543), which correspond to the second binding site of Laura et al. [17] including amino acids: Leu 388, Ala 394, Ala 397, Gln 398, Ile 399, Val 401, Thr 402, Leu 405, Gln 437, Cys 438, Ser 440, Ser 441, Val 442, Ser 443, Ala 444, Val 445, Glu 446, Leu 447, Phe 448, Val 450, Lys 473, Asp 477, Leu 478, Met 481, Arg 482, Pro 485, Ser 486, Ala 517, Ala 520, and Ser 521. In the study of Laura et al., this region is believed to be more selective in binding to the substrates.

The molecular docking model of ABCG2 can be used to analyze the interaction between inhibitors and target. However, the model has some limitations when applied to screening. The model was applied to screen 57,724 substances from the TCM database and 15,464 substances from the DrugBank database. As a result, 714 substances from the DrugBank and 837 substances from the TCM with potential to inhibit the ABCG2 were obtained. 

## 4. Materials and Methods

### 4.1. Data Sources

#### 4.1.1. Databases for Pharmacophore Modelling of ABCG2 Inhibitors

In the study, databases from different studies were collected in order to build ABCG2 inhibitors pharmacophore models (Table 9). After eliminating duplication, there were 375 obtained substances, which were then divided into three groups based on IC_50_ value (pIC_50_) (Table 10). The training set including 15 substances was selected from substances with strong biological activity (IC_50_ value ≤ 1 µM or pIC_50_ ≥ 6), belonging to different skeletal structures and could interact with substrate by a concurrency mechanism (Table S1). The decoy set with 950 substances was generated from 15 strong inhibitors, using the DUDE database (http://dude.docking.org/ accessed date: 17 September 2019). When testing, the mean value would be used in case the biological activity of a substance had many values with the same method. The data from Hoechst 33,342 quantitative method on MDCK II cell line increased ABCG2 expression was chosen because various bioactivity testing on this model had shown an increase in the ABCG2 expression. 

#### 4.1.2. Databases for Virtual Screening

In this work, we utilized 15,464 substances from the DrugBank [12] for in silico screening to seek novel substances might help inhibit ABCG2 pump. Furthermore, 57,424 compounds were also downloaded from Traditional Chinese Medicine (TCM) [13] for the same purpose.

### 4.2. Pharmacophore Modelling

#### 4.2.1. Building Pharmacophore Models

In this study, the conformation of substances were constructed by using Conformation Import tool in MOE 2015.10. The program used algorithms to find the low-energy conformation of substances and save them in a data set. Substances were washed and removed (salts and solvents) by using Wash tool in MOE 2015.10. Input molecules were classified according to the capability to overlap of the fragments. The conformation of fragments was searched randomly and closely. The search results would be saved in a fragmented database to be used for the next time. Conformation of complete molecules were formed by grouping conformations of fragments. The conformation would be removed if the van der Waals interaction is not good or the configuration groups are not suitable. The energy of the complete conformation is the sum of the energy of the fragments created by superposition of 3 atoms or more. Finally, conformation and energy are recorded in the output data. Pharmacophore model was constructed by Pharmacophore Elucidation in MOE 2015.10, which generate pharmacophore queries overlapping well with most of the molecules in the construction set.

#### 4.2.2. Model Evaluation

**Figure 16:**
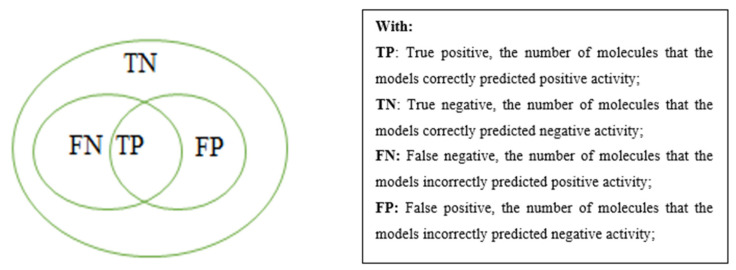
Parameters used for evaluating the pharmacophore model.

The Pharmacophore Search tool was used to re-evaluate the pharmacophore model constructed or to apply pharmacophore for searching for active substances in the application set. Figure 16 describes the parameters used for evaluating the pharmacophore model. 

The decoy set is used to evaluate pharmacophore models by Sensitivity (*Se*), Specificity (*Sp*), Total number of hits (*Ya*), Enrichment factor (*E*), and Gunner–Henry score (*GH* score).

Sensitivity of the model:

$$S_{e}=\frac{TP}{A}=\frac{TP}{TP+FN}$$

Specificity of the model:

$$S_{p}=\frac{TN}{N-A}=\frac{TN}{TN+FP}$$

Ratio of the number of molecules which are correctly predicted to have positive activity to the number of molecules which are predicted to have positive activity (total number of hits):

$$Y_{a}=\frac{TP}{n}=\frac{TP}{TP+FP}$$

Enrichment factor: This index evaluates the ability of the model to give a correct prediction compared to random selection. For example, if a data set has 30% active substances, the model predicts that 30% of hits are active so this is just a random prediction.

$$E=\frac{TP/n}{A/N}$$

The Gunner–Henry “Goodness of hit list” score:

$$GH=\left(\frac{3}{4}Y_{a}+\frac{1}{4}S_{e}\right)S_{p}$$

Gunner–Henry score is used for evaluating the model. The better the model is, the higher the Gunner–Henry score is.

The most selective pharmacophore model was conducted a test on the known biological activity set with 375 substances, including 50 inactive and 325 active substances.

### 4.3. Homology Modelling

#### 4.3.1. Building Homology Models

To construct a homology model of ABCC2, it is necessary to find the amino acid sequence of this protein. The amino acid sequence of the ABCG2 protein was obtained from the website www.uniprot.org (accessed on 17 September 2019) (ID: Q9UNQ0), consisting of 655 amino acids (Supplementary Figure S1). It was then uploaded to the SWISS MODEL server at website https://swissmodel.expasy.org (accessed date: 17 September 2019) [14] to form a homology model [33]. After uploading amino acid sequence to the server, it would automatically find protein samples close to the amino acid sequence of ABCG2 and generate the homology model.

#### 4.3.2. Model Evaluation

The homology model generated by SWISS MODEL server was evaluated through 2 parameters GMQE and QMEAN. QMQE (Global Model Quality Estimation) reflects the quality of the combination of the sample and the generated model, the sample search method. The value of QMQE is from 0 to 1. The higher the QMQE value, the higher the reliability of the model. QMEAN parameter estimates based on different geometries for the entire structure or a particular position of the model and pattern. The closer the QMEAN value to 0, the more reliable the model. The failed model is those with a QMEAN value below −4.

The selected homology model was also stereo-chemical quality tested using PROCHECK Tool [34], in which the structure in PDB format was uploaded into PDBsum to generate its Ramachandran plot. The phi-psi torsion angles of all protein residues were illustrated in the Ramachandran map, except for the chain termini position, where glycine residues were not restricted to any particular region of the plot and were separately identified by triangles. A high quality model would be expected to have over 90% in the core regions, also called the most favored regions [A, B, L].

### 4.4. Molecular Docking

#### 4.4.1. Ligand and Protein Preparation

**Figure 17:**
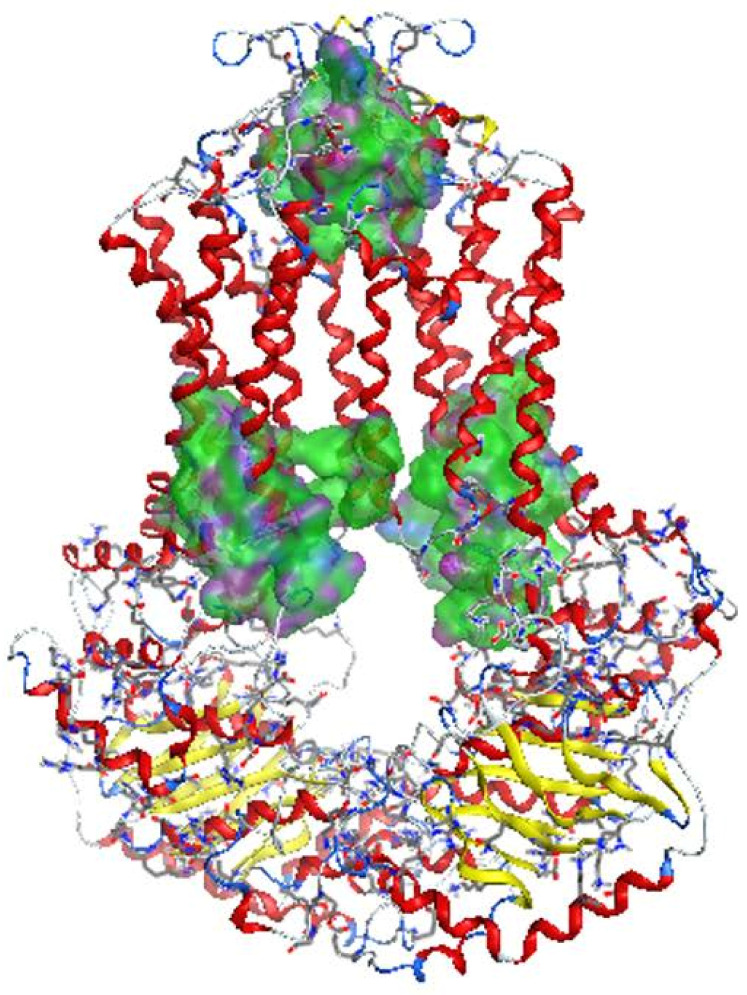
Binding sites determined by Site Finder.

Proteins were prepared by Molecular Operating Environment (MOE) 2015.10 software (Chemical Computing Group, Montreal, QC, Canada) [35] according to the following steps: protonating and charging amino acids (protonate); minimizing energy (Tether and Minimize); converting to *.pdb format. The Site Finder tool in MOE 2015.10 was used to find possible binding sites on the target protein. The most reasonable binding sites were indicated based on experimental data and Site Finder’s results. Figure 17 describes three binding sites on ABCG2 homology structure determined by Site Finder of MOE 2015.10 software.

After the preliminary determination of binding sites, Lead IT 2.3.2 software (BioSolveIt GmbH, Sankt Augustin, Germany) was used for docking ligand to target by Lead IT 2.3.2 [36]. The results were recorded with 10 poses for each compound in order to identify the highest affinity binding site.

Ligands were prepared in order to be stable during docking process. The ligands’ 2D-structures were drawn and transform to 3D structure by ChemBio3D Ultra 13.0 [37]. In *.sdf format, all ligands were energy minimized two times by Sybyl-X 2.0 [38] (Method: Conj Grad; Termination: Energy Change 0.0001 kcal/(mol*A); Max Iterations: 10,000; Charges: Gasteriger-Huckel). After the first minimizing stage, ligands were simulated molecular dynamics by Simulated Annealing tool before the second one. 

#### 4.4.2. Docking

Prepared proteins were upload to Lead IT 2.3.2 software [36]. Binding pocket were selected with radius of 20 Å for fully overlapping all protein surface. Docking process was then per-formed with followed parameters: Number of poses retained = 10, the maximum number of repetitions = 1000, the number of defragments = 200. The docking results were recorded as *.sdf file and read by MOE 2015.10. 

Docking scores (KJ·mol^−1^) were evaluated based on interaction between ligands and protein including ionic bonds, hydrogen bonds, van der Waals, π-π, … The docking results showed the binding affinity of ligands to protein and interaction of ligands with surrounding amino acids, which illustrated inhibitory potential of these substances.

#### 4.4.3. Receiver Operating Characteristic (ROC) Analysis

To validate the docking models, receiver operating curve (ROC) value was analyzed with active and inactive compounds [39]. The decoy set of 950 structures and the active set of 325 structures were docked to ABCG2 protein. As a result, *Se* and *Sp* values were calculated based on docking scores, which were then used for plotting ROC curve (Equation (1)). The area under ROC curve (*AUC*) value was crucial for evaluating the docking models, in which a good model would show ROC nearly to 1.

(1) $$AUC=\overset{n}{\underset{x=2}{\sum }}Se\times [\left(1-Sp\left(x\right)\right)-(Sp\left(x-1\right))]$$

### 4.5. Virtual Screening

**Figure 18:**
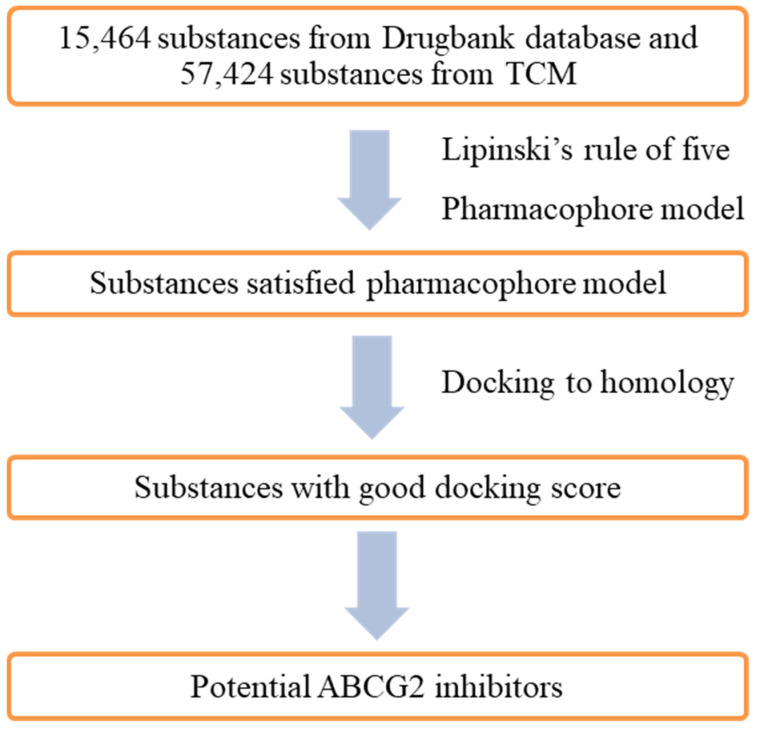
Virtual screening process for ABCG2 inhibitors.

The docking model and pharmacophore model built in this research were used for screening ABCG2 inhibitors from the DrugBank database with 15,464 substances and the Traditional Chinese Medicine (TCM) with 57,424 substances. The query matching compounds was screened by pharmacophore model, while docking process investigated the interactions of these compounds with the target. Potential substances would be priorities to synthesize and test in vitro. The whole virtual screening process for ABCG2 inhibitors was described in Figure 18.

## 5. Conclusions

This research developed two screening models for ABCG2 potent inhibitors, namely pharmacophore models (on ABCG2 potent inhibitors) and molecular docking models (based on the homology model of ABCG2). Pharmacophore models were constructed based on substances with strong ABCG2 inhibitory activity (IC_50_ < 1 μM) with a sensitivity of 73.33% and a specificity of 92.95%. The potent inhibitor models consist of two hydrophobic (Hyd) groups, two hydrogen bonding acceptors (Acc2), and an aromatic or conjugated ring (Aro|PiR). In the study, a homology model of ABCG2 was built from 655 amino acids by server SWISS MODEL with GMQE value = 0.85, the QMEAN value is −2.25 > −4. The model was eligible to conduct molecular docking model. The research also identified the binding regions of ABCG2 inhibitors with important amino acids namely Gln 181, Phe 182, Asn 391, Gln 393, Glu 446, Ser 443, Val 533, Val 534, Val 536, and Leu 539. Three amino acids Asn 391, Gln 393, and Glu 446 formed hydrogen bonds with inhibitors, which correspond to hydrogen bonding acceptors in the pharmacophore model. The remaining amino acids form a binding site with hydrophobic regions. The newly constructed model was applied to dock substances that could inhibit ABCG2 pump. The docking results showed that these substances have a high binding affinity to the binding site (93.54% of the substances have docking score ≤−10 KJ·mol^−1^). Before a better resolution structure of ABCG2 presents, the docking model of this homology protein can be used to predict potential ABCG2 inhibitors.

Regarding in silico testing, the study also used these models for screening ABCG2 inhibitors from DrugBank and TCM databases. The abovementioned models was applied to screen 57,724 substances from the TCM database and 15,464 substances from the DrugBank database. As a result, 714 substances from the DrugBank and 837 substances from the TCM with potential to inhibit the ABCG2 were obtained. Substances with promised results in the study can be priority synthesized or extracted and conducted further tested for bioactivity.

## Figures and Tables

**Table 1:** Properties of two 4-scoring pharmacophore models.

Model	Cover	Overlap Scoring	Accuracy Scoring	Aromatic Ring/Pi Conjugate Ring (Aro/PiR)	Hydrophobic Center (Hyd)	Hydrogen Bonding Acceptor (Acc2)
P1	15	7.7467	1	1	2	1
P2	14	7.0758	0.9333	1	2	1

**Table 2:** Evaluation results of pharmacophore models P1 and P2 by decoy set.

Model	Total Molecules (N)	TP/A	TN/(N-A)	Se (%)	Sp (%)	Ya (%)	Index E	GH Score
RHHa_2	965	15/15	430/950	100	55	3.37	2.1	0.42
RHHa_1	965	14/15	398/950	93.33	58	3.40	2.2	0.41

**Table 3:** Properties of pharmacophore models built from 118 strong inhibitors.

Model	Cover	Overlap Scoring	Accuracy Scoring	Aromatic Ring/Pi Conjugate Ring (Aro/PiR)	Hydrophobic Center (Hyd)	Hydrogen Bonding Acceptor (Acc2)
P3	107	74.7660	0.9068	1	2	1
P4	99	73.7048	0.8390	2	1	1

**Table 4:** Evaluation results of P5 pharmacophore model by decoy set.

Model	Number of molecules (N)	TP/A	TN/(N-A)	Se (%)	Sp (%)	Ya (%)	Index E	GH Score
P5	965	12/15	67/950	73.33	92.95	15.19	9.62	0.55

**Table 5:** The docking results of 15 strong inhibitors.

No.	Name	Docking Score (KJ·mol^−1^)	IC_50_ (nM)
1	JMC_2018_146_483_43	−22.74	61.6
2	JMC_2017_60_4474_47	−20.36	98.8
3	BMC_2013_21_7858_31	−20.09	76
4	JMC_2018_61_3382_15	−18.04	149
5	JMC_2016_117_212_35	−17.8	190
6	CMC_2012_7_650_PD158780	−17.66	360
7	JMC_2009_52_1190_6	−16.91	60
8	JMC_2009_52_1190_Elacridar	−16.55	250
9	JMC_2016_59_6121_51	−16.14	233
10	DMD_2017_45_1166_Curcumin	−15.57	650
11	ACS_2013_4_393_22a	−15.19	591
12	DDDT_2015_9_3481_5j	−13.94	200
13	BMC_2012_20_346_25	−13.07	530
14	JMC_2009_52_1190_Ko143	−6.22	225
15	DMD_2017_45_1166_GOY168	−5.15	250

**Table 6:** Frequency of amino acids interacting with amino acids in the binding site.

No.	Amino Acid	Frequency (Number of Interactions)	Type of Interactions
1	Gln 181	69	Surface interaction; Hydrogen donor interaction
2	Phe 182	197	Surface interaction
3	Asn 391	147	Hydrogen donor, acceptor interaction; Surface interaction
4	Gln 393	262	Hydrogen donor, acceptor interaction; Surface interaction
5	Glu 446	117	Hydrogen donor, acceptor interaction
6	Ser443	96	Surface interaction
7	Val 534	174	Surface interaction
8	Val536	90	Surface interaction
9	Leu 539	282	Surface interaction

**Table 7:** Results docking and model evaluation based on the ROC.

Docking Score (KJ·mol^−1^)	TP	TN	FP	FN	Sp	Se	EF	AUC
≥−28	2	950	323	0	0.75	1.00	3.92	0.92
[−28, −26]	10	950	315	0	0.75	1.00	3.92	
[−26, −24]	30	950	295	0	0.76	1.00	3.92	
[−24, −22]	84	949	241	1	0.80	0.99	3.88	
[−22, −20]	144	938	181	12	0.84	0.92	3.62	
[−20, −18]	187	910	138	40	0.87	0.82	3.23	
[−18, −16]	228	865	97	85	0.90	0.73	2.86	
[−16, −14]	257	758	68	192	0.92	0.57	2.25	
[−14, −12]	287	601	38	349	0.94	0.45	1.77	
[−12, −10]	304	457	21	493	0.96	0.38	1.50	
[−8, −10]	315	322	10	628	0.97	0.33	1.31	
[−8, −6]	324	226	1	724	1.00	0.31	1.21	
[−6, −4]	325	160	0	790	1.00	0.29	1.14	
[−4, −2]	325	124	0	826	1.00	0.28	1.11	
[−2, 0]	325	95	0	855	1.00	0.28	1.08	
<0	325	0	0	950				

**Table 8:** Selected compounds with inhibitory potential for ABCG2.

No.	Name—Common name	Docking scores (KJ·mol^−1^)	Structures
1	DB07256	−29.58	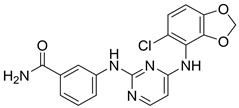
2	DB12186	−26.63	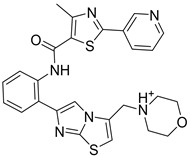
3	DB07253	−25.25	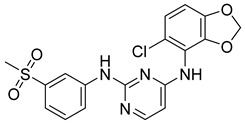
4	DB15009	−24.07	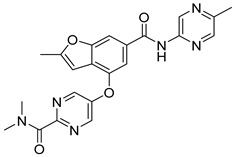
5	DB07845	−23.52	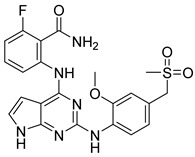
6	DB15448	−23.35	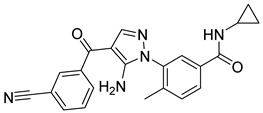
7	DB15310	−22.84	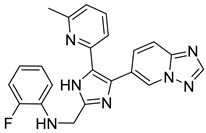
8	DB02089	−22.82	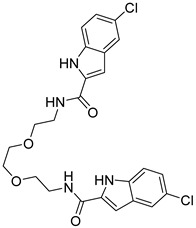
9	DB07586	−22.74	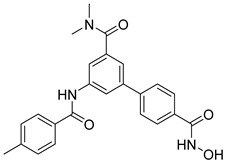
10	DB07006	−22.73	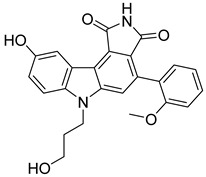
11	TCM_173 Rosmarinic	−22.29	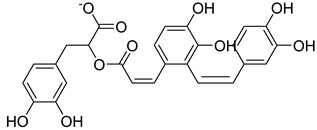
12	TCM_613	−21.53	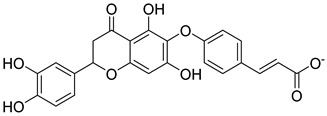
13	TCM_741	−20.59	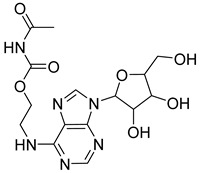
14	TCM_220	−19.84	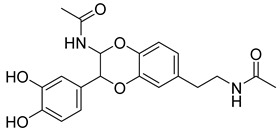
15	TCM_274 Xanthommatin	−19.42	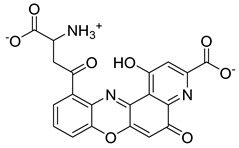
16	TCM_725	−18.99	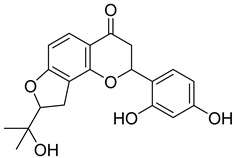
17	TCM_671	−18.99	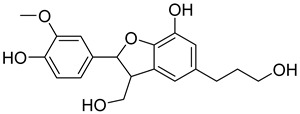
18	TCM_683 Balanophonin	−18.95	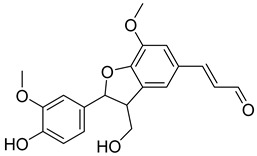
19	TCM_587 6-Prenyleriodictyol	−18.88	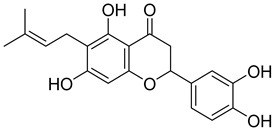
20	TCM_235 Riboflavin	−18.70	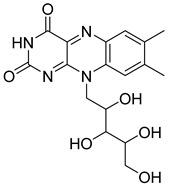

**Table 9:** List of databases used for pharmacophore modelling of ABCG2 inhibitors in this study.

Database	Bioassay Method	Cell Line	Control Substance/IC_50_ (nM)	Number of Tested Activity Substances	Reference
JMC_2015_58_3910	Quantitation Using Hoechst 33,342	MDCK II BCRP	Ko143/128	28	[18]
JMC_2013_67_115	Quantitation Using Hoechst 33,342 and Pheophorbid A	MDCK II BCRP	Ko143/Hoechst 33,342: 215 Pheophorbid A: 354	35	[19]
BMC_2013_21_7858	Quantitation Using Hoechst 33,342 and Pheophorbid A	MDCK II BCRP	Ko143/Hoechst 33342: 250 Pheophorbid A: 330	46	[20]
BMC_2012_20_346	Quantitation Using Hoechst 33,342	MDCK II BCRP và MCF-7	Ko143/MDCK II BCRP: 260 MCF-7: 330	45	[21]
JMC_2018_61_3389	Quantitation Using Hoechst 33,342	MDCK II BCRP	Ko143/227	46	[22]
CMC_2013_8_125	Quantitation Using Hoechst 33,342	MCF-7	-	25	[23]
CMC_2012_7_650	Quantitation Using Hoechst 33,342 and Pheophorbid A	MDCK II BCRP	-	7	[24]
BMC_2012_22_6766	Quantitation Using Hoechst 33,342	MDCK II BCRP	Ko143/250	25	[25]
JMC_2016_59_6121	Quantitation Using Hoechst 33,342	MDCK II BCRP	Ko143/221 XR9577/704	40	[26]
JMC_2018_61_7952	Quantitation Using Hoechst 33,342	MDCK II BCRP	Ko143/227	46	[27]
JMC_2012_55_966	Flow cytometry Mitoxantron	HEK 293-BCRP	-	13	[28]
JMC_2009_52_1190	Flow cytometry Mitoxantron	MCF = 7	FTC tại 10 µM ức chế 100%	15	[29]
JMC_2017_60_4474	Quantitation Using Hoechst 33,342	MDCK II BCRP		38	[30]
JMC_2016_117_212	Quantitation Using Hoechst 33,342	MDCK II BCRP	Ko143/240	23	[31]
DMD_2017_45_1166	Quantitation Using Hoechst 33,342	K562-BCRP	Ko143/190	25	[32]

**Table 10:** Test set of pharmacophore models.

Inhibitory Concentration 50% (IC_50_)	IC_50_ ≤ 1 µM	1 µM < IC_50_ ≤ 10 µM	Ineffective	Decoy Set
Number of substances	155	170	50	950

## Supplementary Materials

The following are available online, Table S1. ABCG2 strong inhibitors in the training set, Table S2. Docking scores of ABCG2 inhibitors in test set of the pharmacophore model, Figure S1. The amino acid sequence of the ABCG2 protein.
