# Supplementary material for: Structure-Based Discovery of ABCG2 Inhibitors: A Homology Protein-Based Pharmacophore Modeling and Molecular Docking Approach
Source: Molecules. 2021 May 23;26(11):3115. doi: 10.3390/molecules26113115 (PMC8197086; doi:10.3390/molecules26113115)
Supplement: Supplementary file 1 [file molecules-26-03115-s001.zip › molecules-1208173-supplementary.pdf]

SUPPORTING INFORMATION

Structure-based discovery of ABCG2 inhibitors: a homology protein-based pharmacophore modeling and molecular docking approach

Minh-Tri Le [1,2], Viet-Nham Hoang [1,3], Dac-Nhan Nguyen [1], Thi-Hoang-Linh Bui [1], Thien-Vy Phan [1,4], Phuong Nguyen-Hoai Huynh [1], Thanh-Dao Tran [1] and Khac-Minh Thai [1,*]

[1] Faculty of Pharmacy, University of Medicine and Pharmacy at Ho Chi Minh City, Ho Chi Minh City 100000, Vietnam; leminhtri@ump.edu.vn (M.-T.L.); hoangvietnham@gmail.com (V.-N.H.); michael.dacnhan@gmail.com (D.-N.N.); bthlinh.duoc16@ump.edu.vn (H.-T.-L.B.); ptvy@ntt.edu.vn (T.-V.P.); huynhnguyenhoaiphuong@gmail.com (P.N.-H.H.); daott@ump.edu.vn (T.-D.T.)

[2] School of Medicine, Vietnam National University Ho Chi Minh City, Ho Chi Minh City 100000, Vietnam

[3] Department of Pharmacy, Lac Hong University, DongNai Province 810000, Vietnam

[4] Department of Pharmacy, Nguyen Tat Thanh University, Ho Chi Minh City 100000, Vietnam

* Correspondence: thaikhacminh@ump.edu.vn or thaikhacminh@gmail.com; Tel.: +84-909-680-385; Fax: +84-28-3822-5435

Table S1. ABCG2 strong inhibitors in the training set

Table S2. Docking scores of ABCG2 inhibitors in test set of the pharmacophore model

Figure S1. The amino acid sequence of the ABCG2 protein

Table S1. ABCG2 strong inhibitors in the training set

No	Substance name/ structure group	Structure	IC_{50} (nM)
**1**	JMC_2009_52_1190_Ko143<br>Derivative of fumitremorgin C	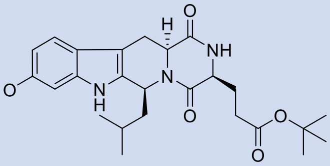	225
**2**	JMC_2009_52_1191_Elacridar<br>Acridone derivative	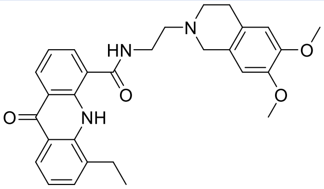	250
**3**	DMD_2017_45_1166_Curcumin<br>Curcumin group	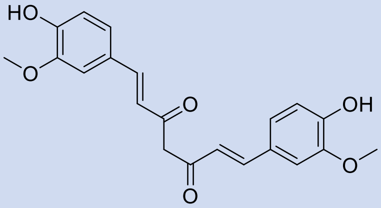	650
**4**	BMC_2012_20_346_25<br>Chalcone derivative	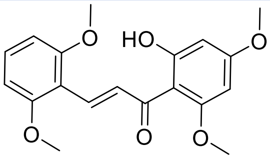	530
**5**	CMC_2012_7_650_PD158780<br>pyrido[3,4,d]pyrimidine derivative	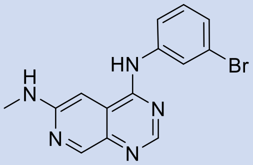	360
**6**	JMC_2009_52_1190_6<br>Tariquidar derivative	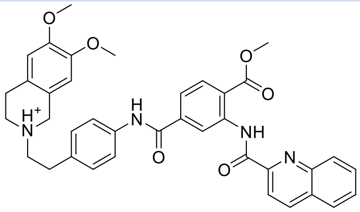	60
**7**	DDDT_2015_9_3481_5j<br>Indeno[1,2-b]indol derivative	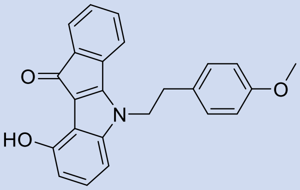	200

No	Substance name/ structure group	Structure	IC_{50} (nM)
**8**	JMC_2016_117_212_35<br>Chalcone derivative combines with quinazoline	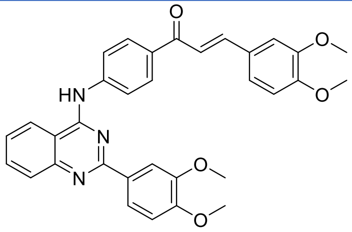	190
**9**	BMC_2013_21_7858_31<br>Quinazoline derivative	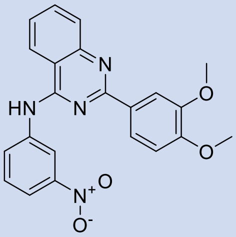	76
**10**	JMC_2018_146_483_43<br>Tariquidar derivative	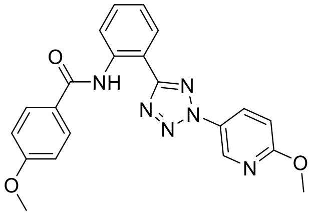	61.6
**11**	JMC_2016_59_6121_51<br>tetrahydro-β-carboline derivative	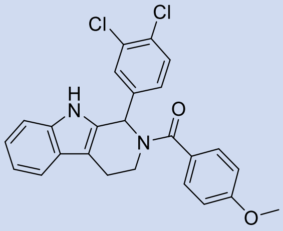	233
**12**	ACSMCL_2013_4_393_22a<br>biphenyl benzanilide derivative	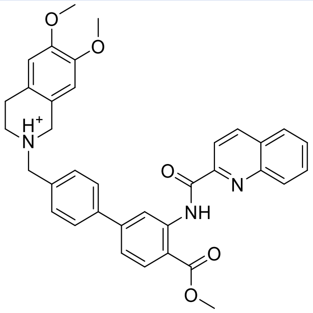	591

No	Substance name/ structure group	Structure	IC_{50} (nM)
**13**	DMD_2017_45_1166_GOY168<br>Curcumin derivative	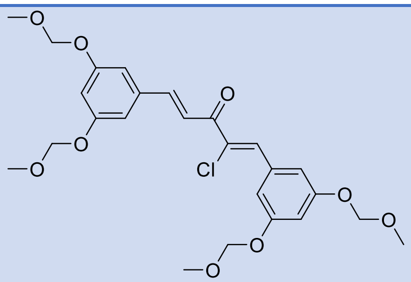	250
**14**	JMC_2018_61_3382_15<br>Pyridopyrimidine derivative	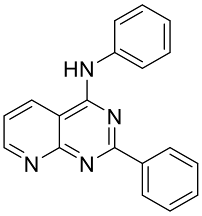	149
**15**	JMC_2017_60_4474_47<br>Pyrimidine derivative	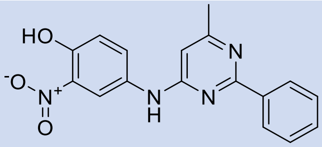	98.8

Table S2. Docking scores of ABCG2 inhibitors in test set of the pharmacophore model

No.	Name	Pharmacophore model	Docking score	pIC_{50}
1	CMC_2015_10_742_14	1	-28.34	5.79
2	BMC_2013_21_7858_53	1	-28.25	6.48
3	CMC_2013_8_125_9	0	-27.56	5.07
4	JMC_2018_146_483_27	1	-27.20	6.57
5	JMC_2018_146_483_37	1	-27.03	6.72
6	JMC_2018_146_483_33	1	-26.99	6.13
7	JMC_2018_146_483_36	1	-26.87	6.59
8	JMC_2018_146_483_30	1	-26.86	6.75
9	CMC_2012_7_650_nilotinib	0	-26.41	6.17
10	JMC_2018_61_7952_49	1	-26.28	5.94
11	JMC_2018_61_7952_24	1	-25.91	7.1
12	CMC_2013_8_125_26	0	-25.90	4.67
13	CMC_2013_8_125_8	0	-25.76	5.41
14	JMC_2018_61_7952_36	1	-25.66	7.18
15	CMC_2013_8_125_7	0	-25.62	5.16
16	JMC_2018_61_3389_22	1	-25.35	5.28
17	JMC_2018_146_483_31	1	-25.28	6.54

No.	Name	Pharmacophore model	Docking score	pIC_{50}
18	JMC_2018_61_7952_39	1	-25.20	7.35
19	JMC_2018_61_7952_16	1	-25.13	6.92
20	JMC_2018_61_7952_57	1	-25.05	6.56
21	JMC_2018_61_3389_34	1	-24.96	5.19
22	BMC_2013_21_7858_22	1	-24.94	5.61
23	CMC_2015_10_742_32	0	-24.89	5.21
24	JMC_2015_58_3910_7	1	-24.63	7
25	CMC_2013_8_125_10	0	-24.53	5.57
26	JMC_2015_58_3910_9	1	-24.46	6.58
27	BMC_2017_22_6766_12	1	-24.44	6.92
28	JMC_2018_61_3389_26	1	-24.33	6.13
29	JMC_2018_61_3389_45	1	-24.12	4.77
30	CMC_2013_8_125_18	0	-24.00	5.09
31	JMC_2018_61_3389_11	1	-23.91	6.28
32	JMC_2018_61_7952_15	1	-23.91	7.19
33	CMC_2015_10_742_7	0	-23.88	5.45
34	JMC_2018_61_7952_25	1	-23.86	6.04
35	CMC_2015_10_742_27	0	-23.81	6.25
36	JMC_2018_61_7952_51	1	-23.68	5.98
37	BMC_2013_21_7858_11	1	-23.66	6.24
38	JMC_2018_146_483_28	1	-23.61	6.37
39	CMC_2013_8_125_16	0	-23.57	5.88
40	BMC_2013_21_7858_26	1	-23.56	6.82
41	JMC_2018_61_7952_41	1	-23.55	7.63
42	JMC_2018_146_483_11	1	-23.54	5.63
43	BMC_2013_21_7858_20	1	-23.51	6.89
44	JMC_2018_61_3389_20	1	-23.42	5.91
45	CMC_2015_10_742_8	0	-23.37	5.75
46	JMC_2018_61_3389_12	1	-23.37	6.28
47	JMC_2018_61_7952_56	1	-23.35	7.1
48	JMC_2018_61_7952_21	1	-23.31	7.29
49	JMC_2015_58_3910_25	1	-23.30	7.1
50	CMC_2015_10_742_21	0	-23.20	5.68
51	JMC_2018_61_3389_44	1	-23.19	5.91
52	BMC_2013_21_7858_14	1	-23.12	5.78
53	JMC_2015_58_3910_11	1	-23.08	6.93
54	JMC_2018_61_7952_37	1	-23.07	7.21
55	JMC_2018_61_3389_30	1	-23.02	6.02
56	JMC_2015_58_3910_10	1	-22.99	7.1
57	JMC_2018_146_483_8	1	-22.94	6.36
58	CMC_2013_8_125_12	0	-22.93	5.59

No.	Name	Pharmacophore model	Docking score	pIC_{50}
59	JMC_2018_61_7952_46	1	-22.89	6.1
60	CMC_2015_10_742_33	0	-22.87	5.47
61	JMC_2018_61_3389_24	1	-22.84	6.79
62	JMC_2018_61_7952_50	0	-22.83	5.79
63	JMC_2018_61_7952_55	1	-22.82	6.45
64	JMC_2018_61_7952_22	1	-22.80	7.31
65	JMC_2018_61_3389_39	1	-22.76	6.21
66	BMC_2013_21_7858_13	1	-22.75	5.71
67	JMC_2018_61_3389_25	1	-22.73	6.34
68	JMC_2018_61_7952_14	1	-22.69	6.97
69	JMC_2018_61_3389_37	1	-22.61	6.79
70	JMC_2015_58_3910_19	1	-22.54	6.57
71	JMC_2018_61_3389_41	1	-22.49	5.82
72	JMC_2018_61_3389_23	1	-22.49	6.75
73	JMC_2015_58_3910_20	1	-22.48	6.7
74	JMC_2018_61_7952_26	1	-22.48	7.33
75	CMC_2013_8_125_11	0	-22.45	5.39
76	JMC_2018_61_3389_40	1	-22.29	6.2
77	JMC_2015_58_3910_22	1	-22.20	7.14
78	BMC_2013_21_7858_24	1	-22.19	6.6
79	JMC_2018_61_7952_31	1	-22.13	7.56
80	JMC_2018_61_3389_46	1	-22.13	6.05
81	JMC_2018_61_3389_10	1	-22.11	6.83
82	JMC_2018_61_7952_30	1	-22.09	7.22
83	JMC_2018_146_483_43	1	-22.06	7.21
84	CMC_2013_8_125_21	0	-22.01	5.46
85	JMC_2018_61_7952_35	1	-22.00	7.35
86	CMC_2015_10_742_24	0	-21.98	5.54
87	JMC_2015_58_3910_23	1	-21.98	6.74
88	JMC_2018_61_3389_38	1	-21.98	6.27
89	JMC_2018_61_7952_58	1	-21.88	5.2
90	JMC_2018_146_483_40	1	-21.83	6.89
91	JMC_2015_58_3910_5	1	-21.82	6.74
92	CMC_2015_10_742_19	1	-21.82	4.64
93	JMC_2018_61_7952_23	1	-21.81	7.43
94	JMC_2018_146_483_12	1	-21.75	4.82
95	CMC_2012_7_650_imatinib	1	-21.73	5.03
96	BMC_2013_21_7858_40	0	-21.68	5.95
97	JMC_2018_61_7952_17	1	-21.67	7.48
98	JMC_2018_61_3389_27	1	-21.64	5.9
99	JMC_2018_61_7952_45	1	-21.59	6.14

No.	Name	Pharmacophore model	Docking score	pIC_{50}
100	JMC_2015_58_3910_14	1	-21.48	6.86
101	JMC_2018_61_3389_21	1	-21.34	5.69
102	CMC_2015_10_742_11	0	-21.32	4.87
103	JMC_2015_58_3910_24	1	-21.30	7.19
104	JMC_2018_146_483_32	1	-21.30	6.35
105	JMC_2015_58_3910_21	1	-21.26	6.93
106	BMC_2013_21_7858_31	1	-21.25	7.12
107	BMC_2013_21_7858_30	1	-21.21	6.19
108	JMC_2018_61_7952_43	1	-21.20	6.43
109	CMC_2015_10_742_20	0	-21.16	5.75
110	CMC_2013_8_125_4	1	-21.16	5.8
111	BMC_2013_21_7858_36	1	-21.12	6.85
112	CMC_2015_10_742_9	0	-21.07	6.03
113	BMC_2012_22_6766_11	1	-21.04	5.23
114	BMC_2013_21_7858_10	1	-21.01	5.43
115	BMC_2013_21_7858_28	1	-20.98	6.54
116	JMC_2018_146_483_10	1	-20.98	6.12
117	JMC_2018_61_3389_35	1	-20.98	6.58
118	CMC_2015_10_742_18	1	-20.97	5.08
119	JMC_2018_146_483_9	0	-20.97	6.95
120	JMC_2018_61_7952_42	1	-20.88	7.24
121	BMC_2013_21_7858_33	1	-20.88	6.52
122	JMC_2018_61_3389_19	1	-20.84	6.69
123	JMC_2018_61_7952_34	1	-20.84	7
124	BMC_2013_21_7858_12	1	-20.82	5.17
125	JMC_2018_146_483_35	1	-20.80	6.57
126	BMC_2013_21_7858_50	1	-20.78	6.17
127	JMC_2018_146_483_24	1	-20.61	5.97
128	JMC_2018_61_7952_32	1	-20.60	7.23
129	CMC_2015_10_742_5	0	-20.54	5.91
130	JMC_2015_58_3910_17	1	-20.51	4.95
131	JMC_2018_61_3389_13	1	-20.48	4.71
132	JMC_2018_61_7952_44	1	-20.44	6.19
133	JMC_2018_61_7952_38	1	-20.41	7.48
134	BMC_2013_21_7858_27	1	-20.41	5.7
135	JMC_2018_146_483_23	1	-20.36	6.54
136	CMC_2015_10_742_17	1	-20.34	5.94
137	CMC_2013_8_125_28	0	-20.29	5.34
138	JMC_2018_61_3389_16	1	-20.28	6.82
139	JMC_2018_61_3389_50	1	-20.24	5.88
140	JMC_2018_61_3389_53	1	-20.18	6.69

No.	Name	Pharmacophore model	Docking score	pIC_{50}
141	JMC_2018_146_483_26	1	-20.14	6.29
142	BMC_2013_21_7858_35	1	-20.14	6.59
143	JMC_2015_58_3910_16	1	-20.13	6.35
144	BMC_2013_21_7858_21	1	-20.00	6.01
145	BMC_2013_21_7858_32	1	-19.98	6.52
146	JMC_2018_61_3389_31	1	-19.86	5.89
147	JMC_2018_61_7952_18	1	-19.82	7.43
148	CMC_2013_8_125_25	0	-19.79	5.94
149	JMC_2016_59_6121_45	0	-19.68	5.88
150	CMC_2013_8_125_13	0	-19.64	5.51
151	JMC_2018_61_7952_48	1	-19.63	5.27
152	JMC_2018_146_483_34	1	-19.63	6.45
153	JMC_2018_146_483_44	1	-19.59	6.99
154	CMC_2015_10_742_16	1	-19.43	5.48
155	JMC_2018_146_483_7	0	-19.40	7.05
156	CMC_2013_8_125_27	0	-19.30	5.44
157	JMC_2018_61_7952_28	1	-19.27	7.12
158	JMC_2016_59_6121_29	0	-19.12	5.11
159	JMC_2018_61_7952_47	1	-19.11	6.08
160	JMC_2018_146_483_25	1	-19.08	6.09
161	JMC_2015_58_3910_18	1	-19.06	6.97
162	JMC_2016_59_6121_40	0	-19.02	5.9
163	BMC_2013_21_7858_47	1	-19.02	6.09
164	JMC_2018_146_483_22	1	-18.95	6.25
165	BMC_2012_20_346_32	0	-18.86	5.01
166	BMC_2013_21_7858_37	1	-18.83	6.33
167	JMC_2016_59_6121_33	0	-18.76	5.96
168	BMC_2012_22_6766_23	1	-18.55	5.1
169	JMC_2018_61_3389_18	1	-18.54	6.46
170	CMC_2013_8_125_17	0	-18.48	5.06
171	JMC_2018_61_3389_52	1	-18.45	6.82
172	JMC_2018_146_483_13	1	-18.42	4.73
173	BMC_2013_21_7858_15	1	-18.42	5.65
174	CMC_2012_7_650_PD158780	0	-18.39	6.44
175	BMC_2012_20_346_20	1	-18.28	5.99
176	BMC_2013_21_7858_17	1	-18.27	5.71
177	BMC_2013_21_7858_51	1	-18.25	6.1
178	JMC_2018_146_483_21	1	-18.19	5.16
179	BMC_2012_20_346_9	0	-18.16	5.71
180	BMC_2013_21_7858_25	1	-18.14	5.82
181	JMC_2018_61_3389_36	1	-18.13	6.37

No.	Name	Pharmacophore model	Docking score	pIC_{50}
182	JMC_2018_146_483_42	1	-18.10	6.89
183	BMC_2012_20_346_37	0	-18.08	4.65
184	CMC_2015_10_742_6	0	-18.06	5.88
185	CMC_2015_10_742_29	0	-18.04	5.74
186	JMC_2018_61_7952_33	1	-18.02	7.52
187	BMC_2012_20_346_34	0	-18.01	5.25
188	BMC_2013_21_7858_38	0	-17.98	5.97
189	JMC_2016_59_6121_41	0	-17.93	5.75
190	JMC_2018_146_483_20	1	-17.91	5.49
191	BMC_2013_21_7858_48	0	-17.84	5.53
192	BMC_2012_20_346_40	1	-17.81	6.14
193	BMC_2013_21_7858_41	0	-17.79	6.03
194	JMC_2018_61_3389_17	1	-17.74	6.62
195	BMC_2013_21_7858_29	1	-17.74	6.38
196	CMC_2013_8_125_23	0	-17.72	4.89
197	JMC_2018_61_3389_32	1	-17.71	6.24
198	JMC_2016_59_6121_35	0	-17.70	6.16
199	JMC_2018_61_3389_33	1	-17.65	6.04
200	BMC_2012_20_346_22	0	-17.42	5.95
201	JMC_2016_59_6121_49	1	-17.39	6.09
202	JMC_2016_59_6121_23	1	-17.39	5.72
203	BMC_2012_22_6766_22	1	-17.23	5.05
204	JMC_2018_146_483_29	1	-17.23	6.64
205	JMC_2016_59_6121_47	1	-17.23	5.84
206	JMC_2018_146_483_41	1	-17.23	7.11
207	JMC_2016_59_6121_31	0	-17.21	5.35
208	JMC_2016_59_6121_26	0	-17.14	5.84
209	BMC_2012_22_6766_24	1	-17.10	5.03
210	CMC_2015_10_742_28	0	-16.94	5.29
211	JMC_2016_59_6121_52	1	-16.87	6.62
212	JMC_2016_59_6121_39	0	-16.77	6.48
213	BMC_2012_20_346_44	0	-16.76	5.14
214	JMC_2016_59_6121_42	0	-16.75	5.99
215	JMC_2016_59_6121_38	0	-16.67	6.13
216	BMC_2013_21_7858_43	1	-16.58	5.76
217	CMC_2012_7_650_AG1428	1	-16.57	5.26
218	BMC_2013_21_7858_46	1	-16.56	5.42
219	JMC_2012_55_966_6i	1	-16.46	6.8
220	JMC_2016_59_6121_32	0	-16.45	5.72
221	BMC_2012_20_346_13	0	-16.43	5.66
222	BMC_2012_20_346_42	0	-16.34	5.25

No.	Name	Pharmacophore model	Docking score	pIC_{50}
223	JMC_2016_59_6121_22	0	-16.31	5.56
224	BMC_2013_21_7858_42	0	-16.09	5.32
225	JMC_2016_59_6121_51	1	-16.08	6.63
226	BMC_2012_20_346_10	0	-16.07	5.48
227	JMC_2016_59_6121_43	1	-16.06	6.16
228	BMC_2012_20_346_41	0	-16.03	5.71
229	BMC_2012_22_6766_19	1	-15.93	5.29
230	BMC_2013_21_7858_44	1	-15.91	5.62
231	CMC_2013_8_125_20	0	-15.84	5.51
232	JMC_2016_59_6121_24	0	-15.78	5.66
233	JMC_2016_59_6121_46	1	-15.72	5.76
234	BMC_2012_22_6766_20	1	-15.71	5.23
235	CMC_2013_8_125_22	0	-15.70	5.66
236	JMC_2018_146_483_19	1	-15.66	5.08
237	JMC_2016_59_6121_53	0	-15.65	6.42
238	BMC_2012_22_6766_21	1	-15.64	5.09
239	JMC_2016_59_6121_37	0	-15.57	6.06
240	JMC_2013_67_115_19	0	-15.44	5.16
241	JMC_2016_59_6121_44	1	-15.42	5.96
242	BMC_2012_20_346_33	1	-15.39	5.71
243	BMC_2012_20_346_31	0	-15.26	5.03
244	JMC_2012_55_966_6f	1	-15.23	6.74
245	CMC_2013_8_125_24	1	-15.21	5.53
246	BMC_2012_20_346_39	0	-15.16	5.54
247	JMC_2012_55_966_6h	1	-15.16	6.7
248	JMC_2013_67_115_21	0	-15.15	4.65
249	BMC_2012_20_346_16	1	-15.11	5.48
250	JMC_2016_59_6121_50	1	-15.03	6.05
251	JMC_2016_59_6121_48	1	-14.99	5.91
252	BMC_2012_22_6766_18	1	-14.98	5.05
253	BMC_2012_20_346_38	0	-14.86	5.59
254	BMC_2012_20_346_5	1	-14.78	5.3
255	JMC_2018_146_483_17	1	-14.76	4.8
256	JMC_2013_67_115_20	0	-14.54	6.14
257	JMC_2016_59_6121_25	1	-14.18	5.44
258	CMC_2012_7_650_gefitinib	0	-13.84	5.49
259	BMC_2012_20_346_14	1	-13.80	5.97
260	BMC_2012_20_346_12	0	-13.77	4.51
261	JMC_2013_67_115_29	0	-13.61	4.96
262	JMC_2016_59_6121_21	0	-13.48	5.63
263	JMC_2016_59_6121_55	0	-13.45	4.98

No.	Name	Pharmacophore model	Docking score	pIC_{50}
264	JMC_2018_146_483_14	0	-13.26	4.4
265	JMC_2013_67_115_17	0	-13.21	6.28
266	JMC_2018_146_483_18	1	-13.17	5.12
267	BMC_2012_20_346_15	0	-13.01	5.83
268	BMC_2013_21_7858_49	1	-12.97	5.86
269	JMC_2013_67_115_16	1	-12.86	5.91
270	JMC_2013_67_115_32	0	-12.84	4.95
271	JMC_2009_52_1191_Elacridar	0	-12.84	6.6
272	JMC_2013_67_115_8	1	-12.82	5.93
273	JMC_2013_67_115_18	0	-12.81	5.54
274	JMC_2012_55_966_6e	1	-12.72	6.33
275	BMC_2012_20_346_21	0	-12.55	5.58
276	BMC_2012_20_346_7	0	-12.55	4.92
277	JMC_2013_67_115_25	0	-12.51	5.61
278	BMC_2013_21_7858_54	1	-12.50	5.76
279	JMC_2012_55_966_6g	1	-12.48	6.96
280	BMC_2012_20_346_11	0	-12.41	5.2
281	JMC_2013_67_115_15	0	-12.40	5.89
282	JMC_2012_55_966_6a	1	-12.37	6.77
283	JMC_2013_67_115_30	0	-12.26	5.05
284	BMC_2012_20_346_30	1	-12.14	5.76
285	BMC_2012_20_346_26	1	-12.11	5.55
286	BMC_2012_20_346_27	1	-12.06	5.94
287	JMC_2013_67_115_26	0	-12.04	5.08
288	JMC_2013_67_115_22	0	-11.90	5.57
289	BMC_2012_20_346_24	1	-11.85	5.52
290	JMC_2013_67_115_24	1	-11.84	6.37
291	JMC_2013_67_115_14	0	-11.83	5.88
292	JMC_2013_67_115_4	0	-11.73	6
293	JMC_2013_67_115_6	0	-11.58	5.92
294	JMC_2013_67_115_27	0	-11.58	5.61
295	JMC_2013_67_115_31	0	-11.57	5.21
296	JMC_2013_67_115_33	1	-11.24	5.51
297	JMC_2013_67_115_28	1	-11.17	6.23
298	JMC_2013_67_115_9	1	-11.07	5.93
299	BMC_2012_20_346_17	0	-10.87	4.97
300	JMC_2013_67_115_23	0	-10.52	5.97
301	BMC_2012_20_346_8	1	-10.36	6.07
302	BMC_2012_20_346_29	1	-10.28	5.11
303	JMC_2013_67_115_7	1	-10.23	5.11
304	JMC_2013_67_115_34	1	-10.00	5.37

No.	Name	Pharmacophore model	Docking score	pIC50
305	BMC_2012_20_346_25	1	-9.99	6.28
306	JMC_2009_52_1191_Tariquidar	1	-9.89	6.04
307	BMC_2012_20_346_28	1	-9.75	5.42
308	JMC_2013_67_115_5	0	-9.67	4.91
309	JMC_2009_52_1191_11	0	-9.35	5.99
310	JMC_2013_67_115_12	1	-9.27	6.27
311	JMC_2009_52_1191_10	0	-8.86	6.2
312	BMC_2012_20_346_19	0	-8.73	5.72
313	JMC_2009_52_1191_14	1	-8.20	6.01
314	JMC_2009_52_1191_15	0	-8.16	5.7
315	JMC_2013_67_115_13	1	-8.13	5.49
316	JMC_2013_67_115_10	1	-7.44	6.09
317	JMC_2009_52_1191_12	0	-6.81	6.5
318	JMC_2013_67_115_11	1	-6.73	5.22
319	JMC_2009_52_1191_5	0	-6.63	6.92
320	JMC_2009_52_1191_9	0	-6.27	6.26
321	JMC_2009_52_1191_8	1	-6.22	6.75
322	JMC_2009_52_1190_Ko143	1	-6.22	6.65
323	JMC_2009_52_1191_7	0	-6.21	6.74
324	JMC_2009_52_1191_6	0	-6.20	7.22
325	JMC_2009_52_1191_13	0	-4.12	6.07

```
>sp|Q9UNQ0|ABCG2_HUMAN ATP-binding cassette sub-family G member 2
OS=Homo sapiens OX=9606 GN=ABCG2 PE=1 SV=3

MSSSNVEVFIPVSQGNTNGFPATASNDLKAFTEGAVLSFHNICYRVKLKSGF
LPCRKPVEKEILSNINGIMKPGLNAILGPTGGGKSSLLDVLAARKDPSGLSG
DVLINGAPRPANFKCNSGYVVQDDVVMGTLTVRENLQFSAALRLATTMTN
HEKNERINRVIQELGLDKVADSKVGTQFIRGVSGGERKRTSIGMELITDPSIL
FLDEPTTGLDSSTANAVLLLLKRMSKQGRTIIFSIHQPRYSIFKLFDSLTLLAS
GRLMFHGPAQEALGYFESAGYHCEAYNNPADFFLDIINGDSTAVALNREED
FKATEIIEPSKQDKPLIEKLAEIYVNSSFYKETKAELHQLSGGEKKKKITVFK
EISYTTSFCHQLRWVSKRSFKNLLGNPQASIAQIIVTVVLGLVIGAIYFGLKN
DSTGIQNRAGVLFFLTTNQCFSSVSAVELFVVEKKLFIHEYISGYYRVSSYFL
GKLLSDLLPMRMLPSIIFTCIVYFMLGLKPKADAFFVMMFTLMMVAYSASS
MALAIAAGQSVVSVATLLMTICFVFMMIFSGLLVNLTTIASWLSWLQYFSIP
RYGFTALQHNEFLGQNFCPGLNATGNNPCNYATCTGEEYLVKQGIDLSPW
GLWKNHVALACMIVIFLTIAYLKLLFLKKYS
```

Figure S1: The amino acid sequence of the ABCG2 protein
